# Evaluation of Electrospun Poly(ε-Caprolactone)/Gelatin Nanofiber Mats Containing Clove Essential Oil for Antibacterial Wound Dressing

**DOI:** 10.3390/pharmaceutics11110570

**Published:** 2019-11-01

**Authors:** Irem Unalan, Stefan J. Endlein, Benedikt Slavik, Andrea Buettner, Wolfgang H. Goldmann, Rainer Detsch, Aldo R. Boccaccini

**Affiliations:** 1 Institute of Biomaterials, Department of Materials Science and Engineering, Friedrich-Alexander-University Erlangen-Nuremberg, Cauerstraße 6, 91058 Erlangen, Germany; irem.unalan@fau.de (I.U.); stefan.endlein@fau.de (S.J.E.); Rainer.Detsch@fau.de (R.D.); 2 Chair of Aroma and Smell Research, Department of Chemistry and Pharmacy, Friedrich-Alexander-University Erlangen-Nuremberg, Henkestraße 9, 91054 Erlangen, Germany; benedikt.slavik@fau.de (B.S.); andrea.buettner@fau.de (A.B.); 3 Institute of Biophysics, Department of Physics, Friedrich-Alexander-University Erlangen-Nuremberg, Henkestraße 91, 91052 Erlangen, Germany; wgoldmannh@aol.com

**Keywords:** electrospinning, PCL, gelatin, clove essential oil, antibacterial, biocompatibility

## Abstract

The objective of this study was to produce antibacterial poly(ε-caprolactone) (PCL)-gelatin (GEL) electrospun nanofiber mats containing clove essential oil (CLV) using glacial acetic acid (GAA) as a “benign” (non-toxic) solvent. The addition of CLV increased the fiber diameter from 241 ± 96 to 305 ± 82 nm. Aside from this, the wettability of PCL-GEL nanofiber mats was increased by the addition of CLV. Fourier-transform infrared spectroscopy (FTIR) analysis confirmed the presence of CLV, and the actual content of CLV was determined by gas chromatography–mass spectrometry (GC-MS). Our investigations showed that CLV-loaded PCL-GEL nanofiber mats did not have cytotoxic effects on normal human dermal fibroblast (NHDF) cells. On the other hand, the fibers exhibited antibacterial activity against *Staphylococcus aureus* and *Escherichia coli.* Consequently, PCL-GEL/CLV nanofiber mats are potential candidates for antibiotic-free wound healing applications.

## 1. Introduction

Microorganisms can quickly enter and instantly grow in open wounds, causing exudate formation, delay in wound healing, and deformation of the skin [1]. Therefore, the most critical issue in the field of wound healing is preventing wound contamination. This drawback could be overcome by using antibacterial wound dressing that protects the area around the wound, providing a suitable moist environment and antibacterial properties [2]. More recently, there has been renewed interest in natural antibacterial agents such as phytotherapeutics for antibiotic-free wound healing applications [3,4]. In particular, essential oils are one of the most promising phytotherapeutics, aiming at the promotion of the wound healing process while minimizing bacterial infections [5,6,7].

Essential oils (EOs) are natural-based compounds that can provide antibacterial, anti-inflammatory, and antioxidation protection [8]. The antibacterial activity of EOs such as clove, cinnamon, oregano, and lemongrass essential oil depends on the chemical structure of their primary component and concentration [9]. Among these, clove essential oil (CLV) has been medicinally used for centuries for its therapeutic effects [10]. CLV is isolated from the aromatic flower buds of *Eugenia caryophyllata*, which is composed of eugenol (78%), β-caryophyllene (13%), and other compounds such as benzyl alcohol [11]. Its primary components have been widely used due to their medicinal properties such as antioxidant, anti-inflammatory, and antimicrobial activities [12]. Moreover, its antibacterial activity has been demonstrated to be particularly effective against bacterial strains such as *Escherichia coli*, *Staphylococcus aureus*, *Bacillus subtilis*, and *Pseudomonas fluorescens* [13]. On the other hand, the effective dose of CLV has enhanced cell viability [14,15]. According to our knowledge, a few studies related to EOs’ influence on in vitro cell migration have been carried out. Aside from this, the application of CLV in wound dressing is limited due to its high volatility and sensitivity to degradation from exposure to oxygen, heat, and light [16]. In order to improve the applicability of CLV, different scaffold preparation methods have been used to develop polymer carriers for CLV, such as casting films [17,18], nanoparticles [19,20] and, more recently, electrospun fiber mats [21].

The electrospinning process is a convenient, versatile, and cost-effective method for fabricating homogeneous and porous macro- or nanofibers [22]. The nanostructure and high surface-area-to-volume ratio of electrospun nanofibers are attractive features to enhance the drug releasing capability for wound healing applications [23]. Poly(ε-caprolactone) (PCL), one of the promising synthetic polymers for wound healing applications, has an excellent processability, good biocompatibility and, compared to natural polymers, high mechanical properties, whereas the biodegradability, cell adhesion, and proliferation responses to PCL are limited [24]. On the other hand, the natural polymer gelatin (GEL) containing molecular components present in the extracellular matrix (ECM) promotes cell adhesion and proliferation properties [25]. Therefore, the combination of PCL and GEL in electrospun fibers should lead to improved mechanical and physical properties coupled with suitable biocompatibility [26]. There are only a limited number of studies reporting the incorporation of natural antimicrobial agents into PCL-GEL electrospun fibers for antibacterial wound dressing applications. For instance, Ramalingam et al. [27] fabricated PCL-GEL hybrid composite mats loaded with *Gymnema sylvestre* for wound dressing applications. Similarly, Fallah et al. [28] developed curcumin (CUR)-loaded electrospun PCL-GEL nanofibers. Their results indicated that the addition of CUR increased the antibacterial activity against both Gram-positive and Gram-negative bacteria [28].

Essential oil incorporated in electrospun fibers for wound dressings are a rather recent technology; therefore, there are no reports on the use of CLV and PCL-GEL for antibacterial wound dressing. In this study, PCL-GEL nanofiber mats containing various concentrations of CLV were fabricated using glacial acetic acid (GAA) as a “benign” (non-toxic) solvent for antibiotic-free wound healing applications. The use of benign solvents in so-called “green” electrospinning has special relevance when using natural products such as EOs [29,30] Here, we investigated the effect of various CLV concentrations (1.5%, 3%, and 6%, *v*/*v*) on morphology, average fiber diameter, contact angle (wettability), cell viability, cell morphology, rate of wound closure, and antibacterial activity. To the best of the authors’ knowledge, the cell migration effect of CLV-loaded PCL-GEL nanofiber mats for wound healing applications has not been investigated before.

## 2. Materials and Methods

### 2.1. Materials

PCL (Mw = 80 kDa), GEL (~300 g Bloom, Type A), CLV (C8392, CAS number: 8000-34-8), standard eugenol (E51791, 99%), and fetal bovine serum (FBS; F2442) were purchased from Sigma Aldrich (Darmstadt, Germany). Glacial acetic acid (GAA; 87003-241) and dichloromethane (DCM; BDH1113-4LG) were obtained from VWR (Darmstadt, Germany). The microorganism strains of *S. aureus* (ATCC25923) and *E. coli* (ATCC25922) were used. Luria/Miller agar (X969.1) and lysogeny broth medium (Luria/Miller, 6673.1) were supplied by Carl Roth GmbH (Karlsruhe, Germany). Dulbecco’s modified Eagle’s medium (DMEM, 31885-023), penicillin/streptomycin (PS, 15140-122) and trypsin/EDTA (25200-056) were purchased from Thermo Scientific (Schwerte, Germany). The normal human dermal fibroblast (NHDF) cell line was obtained from Translation Research Center (TRC) of Friedrich-Alexander-University Erlangen-Nuremberg. All reagents and solvents were of analytical grade.

### 2.2. Fabrication of PCL-GEL/CLV Nanofiber Mats

For the fabrication of PCL-GEL electrospun nanofiber mats, firstly GEL powder (4.8%, *w*/*v*) was dissolved in the GAA (90%, *v*/*v*) solvent at 45 °C for 4 hours. After the GEL was dissolved, PCL pellets (11.2%, *w*/*v*) were added to the solution and stirred overnight at room temperature. The total polymer concentration of GAA solution was fixed at 16% (*w*/*v*). For the preparation of CLV-incorporated solutions, PCL-GEL solution preparation was followed as explained above. After 30 min of addition of PCL pellets, different ratios of CLV (1.5%, 3%, and 6%, *v*/*v*) were separately added in a dropwise manner to the PCL-GEL solution and stirred overnight at room temperature. Then, each electrospinning solution was carried out under defined and constant ambient conditions (temperature (T): 25 °C and relative humidity (RH): 25%) using a commercially available electrospinning setup (IME’s medical electrospinning machines, EC-16 CLI, IME Technologies, Netherlands). The solutions were loaded separately into a 3 mL plastic syringe equipped with a 23G needle and fed at 0.6 mL/h. The aluminum sheet wrapped around the rotating drum collector was placed at a distance of 12 cm from the needle tip to the collector. Electrospinning was conducted by applying a voltage of +19 kV in the needle and −1 kV to the target. Moreover, during the electrospinning process, the gas shield accessory with nitrogen flux was set at 8 mL/min for optimization of the Taylor cone. The samples were then stored at 4 °C in the dark until further analysis.

### 2.3. Characterization of Nanofiber Mats

The surface morphology of nanofiber mats was analyzed by scanning electron microscopy (SEM, ETH: 2 kV, Everhart-Thornley detector (SE2), AURIGA base 55, Carl Zeiss). The samples were coated with a thin layer of gold (Q150T Turbo-Pumped Sputter Coater/Carbon Coater, Quorum Technologies) before SEM analyses. The average fiber diameter of nanofiber mats was measured from the SEM images using the Image J analysis software (NIH, Bethesda, MD, USA). For calculation of the average fiber diameter and fiber distribution, 50 randomly selected different points were measured.

The contact angle meter (Drop Shape Analyzer, DSA 30, CA Measurement setup, Kruess GmbH, Hamburg, Germany) was used to investigate the wettability of nanofiber mats using a sessile drop method. Briefly, the samples were placed on a glass slide before the test and 8 µl of de-ionized water was dropped on the surface of the nanofiber mats. Four measurements were taken at different locations of the same mats and the average value was obtained.

The functional group of PCL-GEL and CLV-loaded PCL-GEL nanofiber mats PCL-GEL/CLV were analyzed by Fourier-transform infrared spectroscopy (FTIR, IRAffinity-1S, Shimadzu). The spectrum analysis was performed with wavenumbers ranging between 400 and 4000 cm^−1^ and at a spectral resolution of 4 cm^−1^.

The investigation of the total CLV content in CLV-loaded PCL-GEL nanofiber mats was performed using gas chromatography–mass spectrometry (GC-MS, GC/MSD Systems, GC 7890A, MSD 5975C, Agilent Technologies, Waldbronn, Germany) equipped with a DB-FFAP (Durabond-Free Fatty Acid Phase) capillary column (GC Column, 30 m × 0.25 mm, film thickness 0.25 µm; Agilent Technologies, Santa Clara, CA). Briefly, the calibration curve of eugenol, that is, the main component in the volatile fraction of CLV, was prepared and the eugenol concentration was calculated using the peak area (*y* = 77732*x* + 89964, R*^2^*= 0.9995). On the other hand, the nanofiber mats (3 mg) were dispersed in DCM (20 mL) for 2 h. Subsequently, the solvent-assisted flavor evaporation technique (SAFE) [31] was used for the isolation of the CLV volatile fraction. The volatile fraction in DCM was dried over sodium sulfate and then filtered. The final volume (100 µL) was obtained using Vigreux and microdistillation at 50 °C [32]. Then, 1 µL of each sample was taken for the GC-MS measurements. The oven temperature was programmed at 40 °C for 2 min and then heated up at 8 °C/min to 240 °C and held for 5 min. Helium was used as carrier gas at a flow rate of 1 mL/min. Mass spectra were recorded in selected ion monitoring (SIM) mode (*m*/*z* ratio 164). Finally, the percentage of encapsulation efficiency was calculated as Equation (1):

(1) $$\mathrm{Encapsulation}\;\mathrm{Efficiency}\;\left(\mathrm{EE}\right)\left(\%\right)=\frac{M_{a}}{M_{t}}\;\times \;100$$

where M_a_ and M_t_ are the actual and theoretical amounts of CLV in nanofiber mats, respectively.

### 2.4. Antibacterial Assay

The antibacterial activity of CLV-loaded PCL-GEL nanofiber mats was separately tested with *S. aureus* (Gram-positive) and *E. coli* (Gram-negative) bacteria. Initially, the bacterial suspensions were prepared for both bacteria stains in lysogeny broth medium at 37 °C for 24 h. Then, the optical density (OD) (600 nm, Thermo Scientific GENESYS 30, Germany) of the cultivated bacteria was arranged at 0.015. The nanofiber mats (~3 mg) were then cut and were sterilized by UV light irradiation for 30 min. The samples were immersed in lysogeny broth medium, and 20 µL of bacteria suspension was added. Finally, all the samples were incubated at 37 °C for 3, 6, and 24 h and then they were measured at each time interval at 600 nm OD. The relative viability of the bacteria was calculated according to Equation (2):

(2) $$\mathrm{Relative}\;\mathrm{viability}\;\left(\%\right)=\frac{{\mathrm{OD}}_{\mathrm{Sample}}}{{\mathrm{OD}}_{\mathrm{Control}}}\;\times 100$$

The lysogeny broth medium and bacterial cell suspension in lysogeny broth medium were used as a blank and control (CNT), respectively. The experiments were performed three times.

### 2.5. In Vitro Assay

The cell viability, cell morphology, and wound closure rate of NHDF cells were analyzed to gain the first impression of the biological behavior of PCL-GEL, PCL-GEL/CLV1.5, PCL-GEL/CLV3, and PCL-GEL/CLV6 nanofiber mats.

#### 2.5.1. Cell Culture

NHDF cells were cultured in DMEM supplemented with 10% FBS and 1% penicillin/streptomycin in 75 cm^2^ cell culture flasks (Nunc, Denmark). Subsequently, NHDF cells were grown to confluency, and then the cells were detached by trypsinization and counted by trypan blue assay using a hemocytometer (Roth, Germany). Counted cells were seeded in 24-well plates at a density of 50.000 cell/well and incubated at 37 °C in a humidified incubator with 5% CO_2_ for 24 h. Prior to cell culture experiments, the nanofiber mats were fixed on CellCrown 24 inserts (ScaffdexOy, Tampere, Finland) and sterilized by UV light irradiation for 30 min. After 24 h incubation, the medium of the cell was refreshed, and the samples were immersed in a 24-well plate without touching the cells. Finally, the cells were incubated with samples for a further 48 h.

#### 2.5.2. Cell Viability

The viability of the cells was analyzed by a WST-8 cell counting assay kit (Sigma Aldrich). The basis of this assay is the reduction of yellow-colored tetrazolium salt in WST-8 to form orange formazan crystals by dehydrogenases enzymes secreted by mitochondria of metabolically active cells. The amount of these formazan crystals is directly proportional to the number of living cells. After culturing the cells for 48 h, they were washed with PBS to remove unattached cells and incubated with 5% WST-8 reagent in DMEM for a period of 2h at 37 °C. The absorbance of the dye was measured at 450 nm using a spectrophotometric plate reader (PHOmo, anthos Mikrosysteme GmbH, Germany). The percentage of cell viability was calculated as follows:

(3) $$\mathrm{Cell}\;\mathrm{viability}\;\left(\%\right)\;=\frac{\left(\mathrm{Absorbance}\;\mathrm{of}\;\mathrm{control}\;-\;\mathrm{Absorbance}\;\mathrm{of}\;\mathrm{test}\;\mathrm{sample}\right)}{\left(\mathrm{Absorbance}\;\mathrm{of}\;\mathrm{control}\;-\;\mathrm{Absorbance}\;\mathrm{of}\;\mathrm{blank}\right)}\;\times \;100$$

All samples were measured in triplicate.

#### 2.5.3. Hematoxylin and Eosin (H&E) Staining

Hematoxylin and eosin (H&E) staining methods were used for analyzing the cell density. Briefly, the cells were washed with PBS and fixed with Fluoro-Fix for 15 min. After that, the cells were stained with Hematoxylin for 20 min and washed with tap water followed by Scott’s tap water for 5 min and then further rinsed with de-ionized water. After hematoxylin dying, the cells were stained with 0.4% eosin stain (in a saturated aqueous solution of 60% ethanol and 5% acetic acid) for 5 min. Cells were dehydrated with 95% and 100% ethanol, respectively, and air-dried in a fume hood. Finally, the cell density of the NHDF cells was examined using a light microscope (Primo Vert, Carl Zeiss).

#### 2.5.4. In Vitro Wound Healing Assay (Scratch Test)

**Figure 1:**
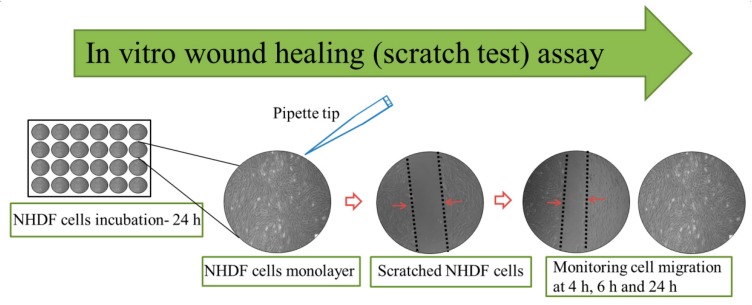
Schematic illustration of in vitro wound healing assay (scratch test) set-up for the study. NHDF: normal human dermal fibroblast.

In vitro healing assay was performed according to the procedure described in [33]. The experimental set-up for the scratch test is schematically illustrated in Figure 1. Firstly, NHDF cells were seeded at 50,000 cell/well into 24-well plate and were incubated at 37 °C in 5% CO_2_ for 24 h. Then, a vertical scratch was created using a 1000 mL sterile pipet tip in the middle of the NHDF monolayer. Subsequently, the nanofiber mats were fixed on CellCrown 24 inserts (ScaffdexOy, Tampere, Finland) and placed in the 24-well plate without touching the surface. Finally, the wound closure rate was monitored at different times (0 h, 4 h, 6 h, and 24 h) and photographed using a light microscope (Primo Vert, Carl Zeiss). The images were analyzed with Image J (NIH, Bethesda, MD, USA). The rate of wound closure was calculated using the following formula:

(4) $$\mathrm{Rate}\;\mathrm{of}\;\mathrm{wound}\;\mathrm{closure}\;\left(\%\right)=\frac{\left(A_{0}-A_{t}\right)}{A_{0}}\;\times \;100$$

where A_0_ is the initial wound area and A*_t_* is the wound area at the designated time.

### 2.6. Statistical Analysis

Data were analyzed by ANOVA and Bonferroni’s test using the Origin (OriginLab, Northampton, MA, USA). The level of significance was determined as *p* < 0.05.

## 3. Results

### 3.1. Surface Morphology of PCL-GEL/CLV Nanofiber Mats

**Figure 2:**
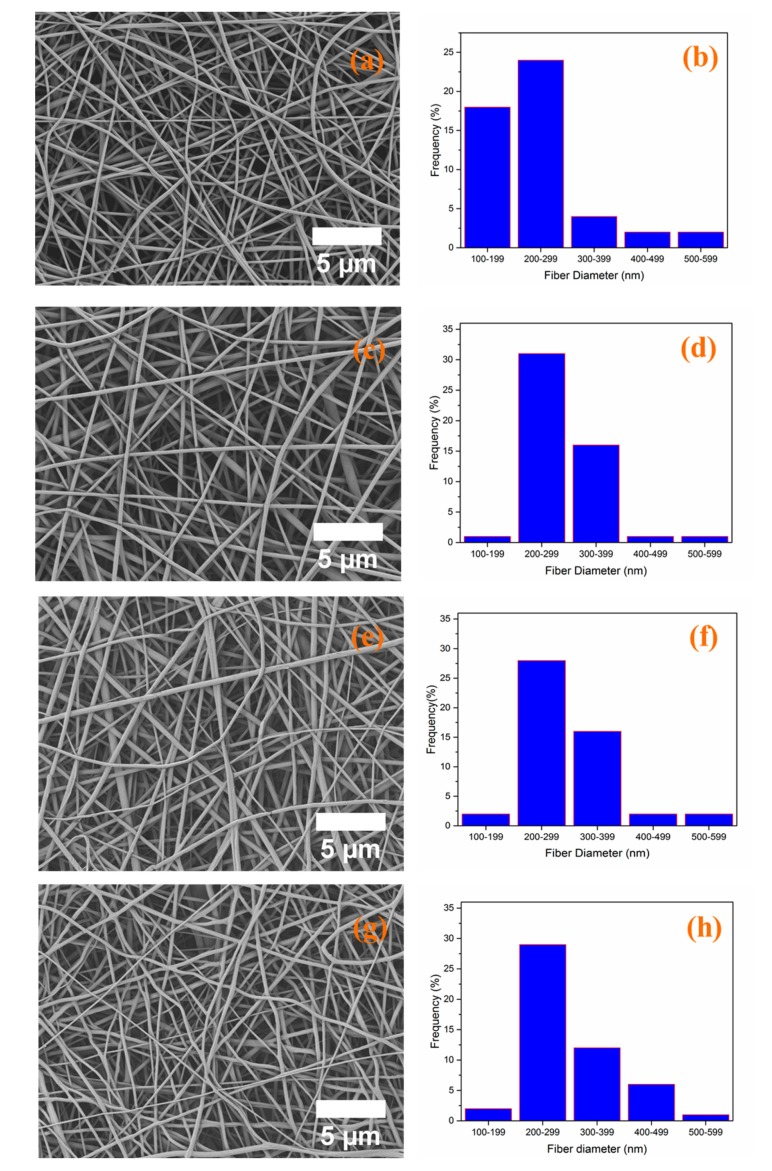
The morphology and fiber diameter distribution of poly(ε-caprolactone) gelatin (PCL-GEL) (**a**,**b**), PCL-GEL/clove essential oil (CLV)1.5 (**c**,**d**), PCL-GEL/CLV3 (**e**,**f**), and PCL-GEL/CLV6 (**g**,**h**) nanofiber mats, respectively.

The morphology of PCL-GEL, PCL-GEL/CLV1.5, PCL-GEL/CLV3, and PCL-GEL/CLV6 nanofiber mats was investigated. The results revealed that all samples had a smooth, uniform, and bead-less morphology. Figure 2 and Table 1 show the fiber distribution and average fiber diameter, separately. The average fiber diameters were 241 ± 96, 285 ± 67, 300 ± 73, and 305 ± 82 nm for PCL-GEL, PCL-GEL/CLV1.5, PCL-GEL/CLV3, and PCL-GEL/CLV6, respectively. The results indicate that the fiber diameter had increased with the addition CLV compared to PCL-GEL.

### 3.2. Wettability

The wettability of the nanofiber mats was analyzed by the sessile drop method. The contact angle values of the samples are shown in Table 1. The incorporation of CLV within the PCL-GEL nanofiber mats resulted in a decreasing contact angle compared to the PCL-GEL nanofiber mats. The contact angle values were 37 ± 8°, 18 ± 3°, 21 ± 4°, and 27 ± 5° for PCL-GEL, PCL-GEL/CLV1.5, PCL-GEL/CLV3, and PCL-GEL/CLV6 nanofiber mats.

### 3.3. Fourier-Transform Infrared Spectroscopy Analysis

**Figure 3:**
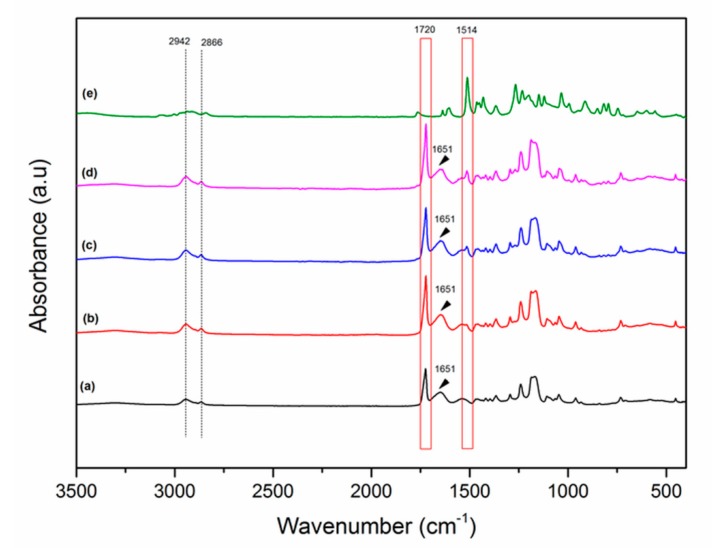
The Fourier-transform infrared spectroscopy (FTIR) spectra of PCL-GEL (**a**), PCL-GEL/CLV1.5 (**b**), PCL-GEL/CLV3 (**c**), and PCL-GEL/CLV6 (**d**) nanofiber mats and pure CLV (**e**).

The presence of CLV within the nanofiber mats was analyzed by Fourier-transform infrared spectroscopy (FTIR) (Figure 3). The typical peaks of PCL were at 2942 and 2866 cm^−1^, corresponding to the asymmetric and symmetric stretching of CH_2_ bonds. Further peaks were at 1720, 1240, and 1168 cm^−1^ related to carbonyl stretching as well as both asymmetric and symmetric stretching of C–O–C bonds, respectively [30]. Characteristic GEL peaks were obtained at 1651 cm^−1^ for amide I and N–H deformation at around 1540 cm^−1^ for the amide II [34]. With the addition of CLV, a new peak appeared within the nanofiber mats. The CLV bands were observed at 1514 cm^−1^, which corresponds to the main component of CLV—eugenol—attributed to the stretching vibration of C=C aromatic bonds [17].

### 3.4. CLV Content in PCL-GEL Nanofiber Mats

The encapsulation efficiency of CLV in PCL-GEL nanofiber mats was investigated by GC-MS. The effect of the different concentrations of CLV on the percentage of encapsulation efficiency is shown in Table 1. The encapsulation efficiency of PCL-GEL/CLV3 and PCL-GEL/CLV6 nanofiber mats were higher than the PCL-GEL/CLV1.5. However, there were no statistically significant differences between the PCL-GEL/CLV3 and PCL-GEL/CLV6.

### 3.5. Antibacterial Assay

**Figure 4:**
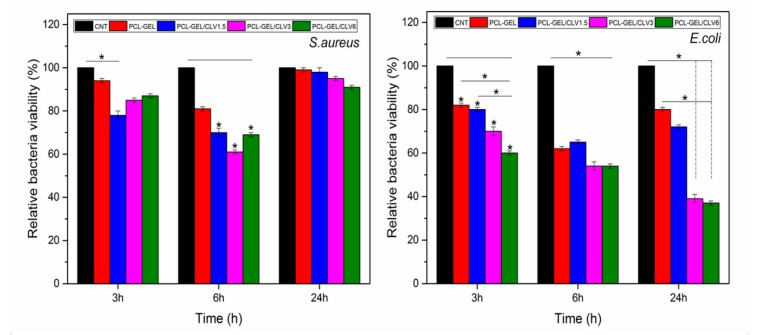
Antibacterial activity of PCL-GEL and CLV-loaded PCL-GEL nanofiber mats after 3, 6, and 24 h incubation with *S. aureus* (Gram-positive) and *E. coli* (Gram-negative) bacteria (*n* = 3, samples in triplicate,* *p* < 0.05).

The antibacterial activity of PCL-GEL, PCL-GEL/CLV1.5, PCL-GEL/CLV3, and PCL-GEL/CLV6 nanofiber mats was tested with *S. aureus* (Gram-positive) and *E. coli* (Gram-negative) bacteria. The bacterial viability against both bacteria strains is illustrated in Figure 4. CLV addition improved the antibacterial activity of nanofiber mats compared to PCL-GEL. The highest inhibition effect was observed at 6 h for *S. aureus,* whereas *E. coli* was active up to 24 h. However, there were no significant differences between the PCL-GEL/CLV1.5, PCL-GEL/CLV3, and PCL-GEL/CLV6 nanofiber mats. Moreover, after 24 h incubation, PCL-GEL/CLV3 and PCL-GEL/CLV6 showed lower *E. coli* bacteria viability than the control (CNT) and PCL-GEL. On the other hand, bacteria viability of *S. aureus* PCL-GEL/CLV1.5, PCL-GEL/CLV3, and PCL-GEL/CLV6 nanofiber mats were reduced compared to CNT at 6 h incubation.

### 3.6. In Vitro Assay

#### 3.6.1. Cell Viability

**Figure 5:**
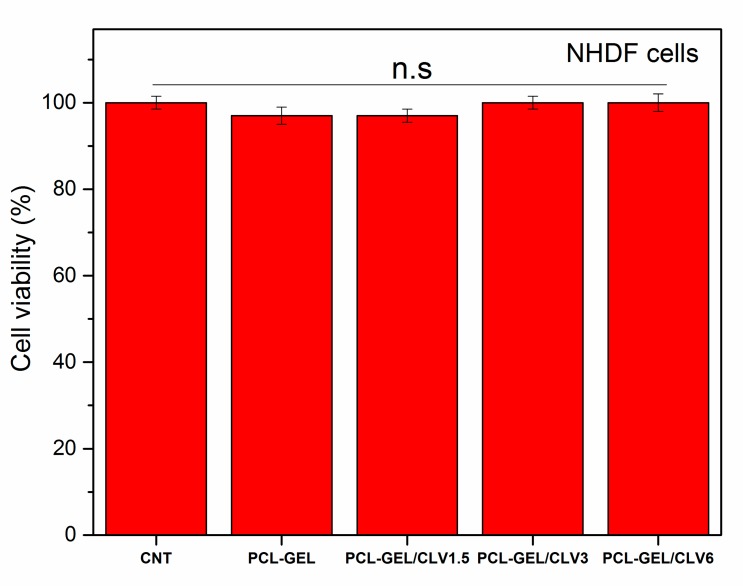
Cell viability percentage of PCL-GEL and CLV-loaded PCL-GEL nanofiber mats after 48 h incubation (*n* = 6, n.s: not significant).

The viability and growth of NHDF cells were determined to investigate the in vitro biocompatibility of PCL-GEL, PCL-GEL/CLV1.5, PCL-GEL/CLV3, and PCL-GEL/CLV6 nanofiber mats after 48 h of incubation, as shown in Figure 5. It is shown that there was no apparent difference between the nanofiber mats compared to the control (CNT). Furthermore, there was no significant change in NHDF cell viability with the increase of to CLV concentration in the nanofiber mats (*p* < 0.05). The results demonstrated that PCL-GEL and PCL-GEL/CLV nanofiber mats show no cytotoxicity on NHDF cells.

#### 3.6.2. Hematoxylin and Eosin (H&E) Staining

**Figure 6:**
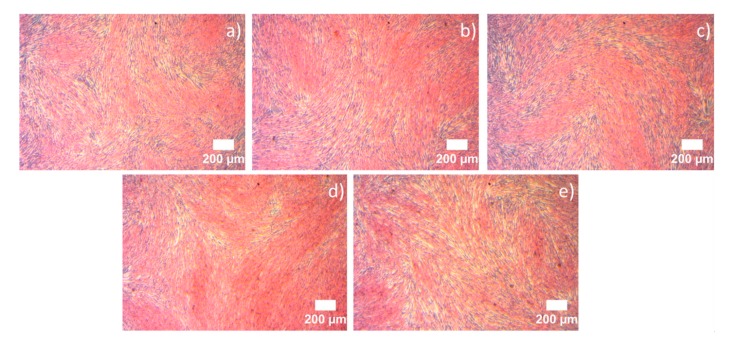
Light microscopy images of hematoxylin and eosin (H&E)-stained NHDF cells cultured with CNT (**a**), PCL-GEL (**b**), PCL-GEL/CLV1.5 (**c**), PCL-GEL/CLV3 (**d**), and PCL-GEL/CLV6 (**e**) nanofiber mats. The H&E staining highlighted the cell nuclei (blue) within the pink cytoplasm.

Light microscopy images of H&E-stained NHDF cells cultured with PCL-GEL, PCL-GEL/CLV1.5, PCL-GEL/CLV3, and PCL-GEL/CLV6 nanofiber mats after 48 h are shown in Figure 6. As evident from light microscope images, the cells exhibited phenotypical cell morphology and were firmly attached and well-spread on the well plate. Also, the addition and increasing the CLV did not affect the morphology and spreading of the NHDF cells. Moreover, the cell confluence obtained after each treatment was in agreement with cell viability measured by the WST-8 assay.

#### 3.6.3. In Vitro Wound Healing Assay (Scratch Test)

**Figure 7:**
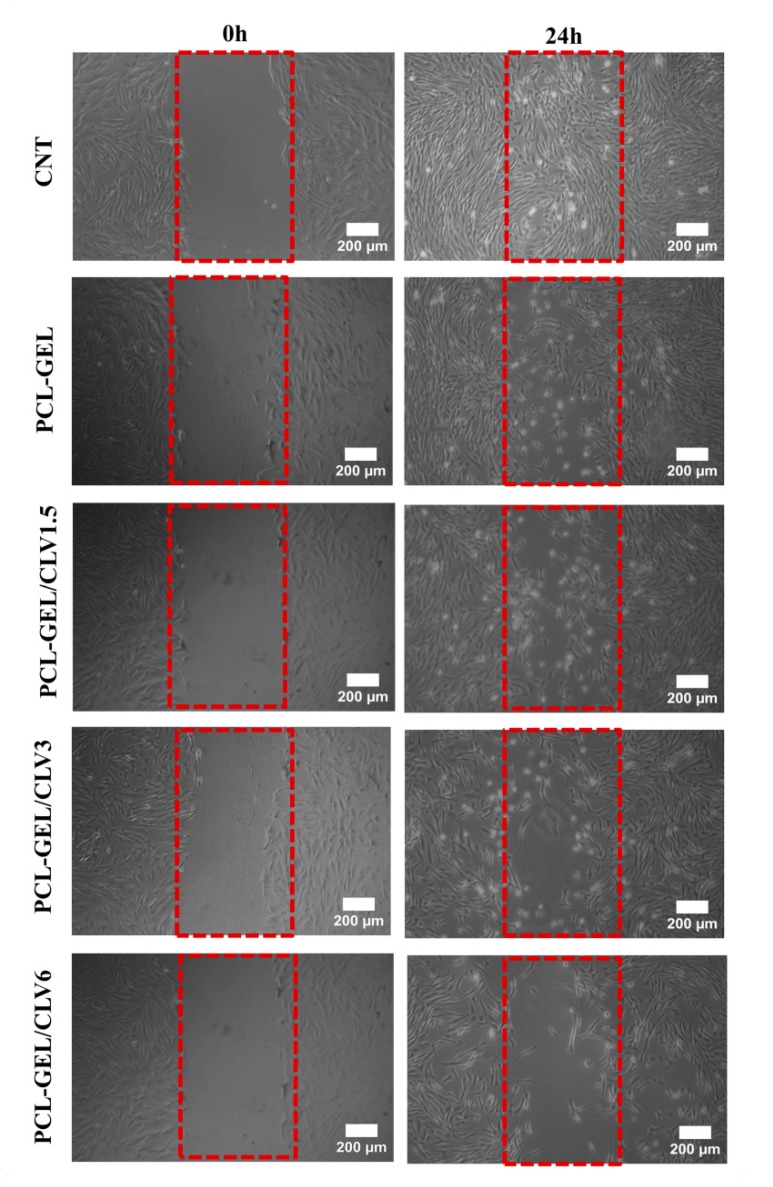
Illustrative micrographs of cell migrating into a scratch area over a 24 h period in CNT, PCL-GEL, PCL-GEL/CLV1.5, PCL-GEL/CLV3, and PCL-GEL/CLV6.

**Figure 8:**
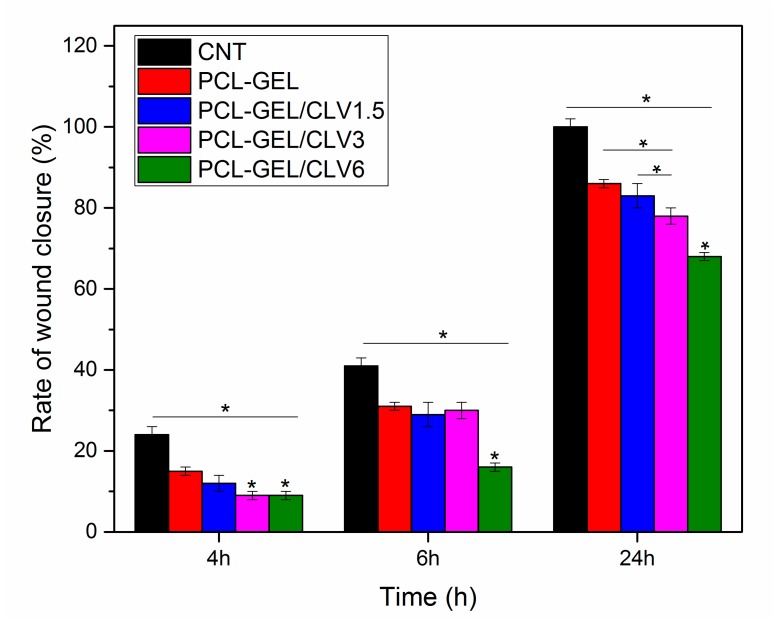
In vitro wound healing assay in the presence of CNT, PCL-GEL, PCL-GEL/CLV1.5, PCL-GEL/CLV3, and PCL-GEL/CLV6 in 24 h (*n* = 3, samples in triplicate, * *p* < 0.05).

The effect of CLV-loaded into PCL-GEL nanofiber mats to the wound healing activity of NHDF cells was assessed using the in vitro wound healing (scratch) assay. The cell migration into the wound areas and rate of wound closure are presented in Figure 7 and Figure 8, respectively. After 6 h, the percentages of cells in the wound area for the control (CNT), PCL-GEL, PCL-GEL/CLV1.5, PCL-GEL/CLV3, and PCL-GEL/CLV6 nanofiber mats were 41 ± 1, 31 ± 1, 29 ± 3, 30 ± 2, and 16 ± 1%, respectively (Figure 8). However, after 24 h of treatment, in the CNT group complete closure was observed, whereas in PCL-GEL, PCL-GEL/CLV1.5, PCL-GEL/CLV3, and PCL-GEL/CLV6 nanofiber mats, this closure was 86 ± 1, 83 ± 3, 78 ± 2, and 68 ± 1%, respectively. Increasing CLV concentration inhibited NHDF cell migration and proliferation in a dose-dependent manner. On the other hand, there was no significant (*p* < 0.05) difference in wound closure rates between PCL-GEL and PCL-GEL/CLV1.5 at 4, 6, and 24 h.

## 4. Discussion

The usage of EOs for antibacterial wound dressing is attracting increasing interest due to their antibacterial, anti-inflammatory, and antioxidant properties. In this study, CLV-loaded PCL-GEL nanofiber mats were produced using benign solvents for antibacterial wound dressings. The concentrations used in the fabrication of nanofiber mats are shown in Table 1. SEM images of the obtained nanofiber mats with and without CLV are depicted in Figure 2. The results indicate that the incorporation of CLV did not affect the fiber formation. However, the average fiber diameter increased by the addition of CLV and increased the CLV amount from 1.5% to 6% (*w*/*v*). It is well known that the fabrication of uniform homogenous, smooth, and bead-free nanofiber morphology depends on electrospinning parameters such as polymer concentration, solution viscosity, electrical conductivity, temperature, and humidity [22]. The lower electrical conductivity of the solution affects the elongation of the jet by the electrical forces and can cause large fiber diameters [35]. The increment of the PCL-GEL/CLV nanofiber diameter could be attributed to the reduction of solution electric conductivity. Aside from this, increasing the solution viscosity led to increased average fiber diameter [36]. In the present study, the addition of CLV could reduce the solution viscosity due to the interaction between CLV and PCL-GEL. In a similar study, García-Moreno et al. [37] reported that the average fiber diameter of poly(vinyl alcohol) (PVA) nanofibers was increased with the encapsulation of fish oil. Mori et al. [38] showed similar results in that various concentrations of candeia essential oil addition increased the average fiber diameter of polylactic acid (PLA) nanostructured mats.

The contact angle measurement, which is a cost-effective and useful method used to investigate the effect of additives on surface properties, has been commonly used to demonstrate the wettability of biomaterials. Furthermore, the increment of the surface wettability enhances the absorption of excess wound exudates, which is important for wound healing applications [39]. In this study, the contact angle measurement was used to determine the effect of CLV on the PCL-GEL nanofiber mats (Table 1). Data obtained in this study indicated that the wettability of PCL-GEL nanofiber mats was increased with CLV addition. This result could be due to the chemical structure of CLV that presents polar phytochemicals and hydrophilic –OH groups. However, increasing the concentrations of CLV, the contact angle of PCL-GEL/CLV1.5, PCL-GEL/CLV3, and PCL-GEL/CLV6 nanofiber mats was slightly increased, which might be due to the loss of free functional groups (amino and hydroxyl groups) on the GEL. According to Liu et al. [40], cinnamon essential oil encapsulated chitosan nanoparticle incorporation in poly (lactic acid) (PLA) composite fibers, increasing the hydrophilic behavior. In another study, Anges Mary and Giri Dev [41] fabricated PCL electrospun matrices incorporated with aloe vera (AV). Their results revealed that the addition of AV decreased the contact angle value compared to pure PCL [41]. Similarly, findings of Ramalingam et al. [27] indicated that PCL/GEL hybrid composite mats comprising natural herbal extract (*Gymnema sylvestre*) had lower contact angle value.

FTIR analysis (Figure 3) revealed the presence of CLV loading in the PCL-GEL nanofiber mats, attributable to the characteristic peak of CLV.

The influence of different CLV ratios in PCL-GEL nanofiber mats on the encapsulation efficiency was examined (Table 1). On the basis of the findings in this study, increasing the CLV content from 1.5% to 3% (*v/v*) increased the encapsulation efficiency. However, the encapsulation efficiency of PCL-GEL/CLV3 and PCL-GEL/CLV6 nanofiber mats were not significantly different. This result could have been due to the capacity of nanofibers, which exhibited a maximum encapsulation efficiency of 73 ± 3%. Recently, clove oil (CO)/chitosan nanoparticles embedded gelatin nanofibers were produced by Cui et al. [20]. The encapsulation efficiency results revealed values that measured ranged from 21.1 ± 0.4% to 39.6 ± 0.8%. Similarly, Tampau et al. [42] reported the fabrication of starch or PCL-based matrices incorporated with carvacrol. The results showed that the encapsulation efficiency of carvacrol in starch or PCL-based matrices increased from 15% to 75% with increasing carvacrol ratios. Moreover, gelatin nanofiber mats encapsulated with orange essential oil were studied by Tavassoli-Kafrani et al. [43] They found that the increment of the orange essential oil ratio in the gelatin nanofibers increased the encapsulation efficiency from 35 ± 0.09% to 69.0 ± 0.22%.

The antibacterial properties of CLV have been investigated using various bacteria such as *E. coli*, *S. aureus*, *Bacillus subtilis*, and *Pseudomonas fluorescens* [13]. The present study investigated the antibacterial activity of CLV-loaded PCL-GEL nanofiber mats against *S. aureus* as Gram-positive bacteria and *E. coli* as Gram-negative bacteria for antibacterial wound dressings. The results indicated that the addition of CLV reduced the bacteria viability for both bacteria strains, as shown in Figure 4. The lowest bacterial viability was observed at 6 h for *S. aureus*, whereas bacterial viability of *E. coli* was reduced at 24 h. This result could have been due to different bacterial characteristics and morphology; for instance: Gram-negative bacteria have a thicker double layer cell membrane than the single membrane of Gram-positive bacteria [5,12]. In a similar study, Fallah et al. [28] reported that the incorporation with curcumin exhibited antibacterial activity against both Gram-positive and Gram-negative bacteria on PCL-GEL fiber mats. Similarly, Figueroa-Lopez et al. [44] produced GEL-coated PCL ultrathin fibers containing black pepper oleoresin (OR). Their results revealed that the viability of *S. aureus* was reduced with the addition of OR. In another study, Ramalingam et al. [27] evaluated the effect of the addition of *Gymnema sylvestre* as a natural herbal in PCL-GEL on the antibacterial performance of these nanofibrous mats. It was found that the antibacterial activity of the samples all increased with the addition of *Gymnema sylvestre*. Furthermore, our results revealed that the highest bacteria inhibition was observed in *E. coli* compared to *S. aureus*. According to Li et al. [45], eugenol loaded PCL-GEL fiber mats are more effective against *S. aureus* compared to *E.coli*; this is in contrast to our studies. The result might have been due to the chemical structure of CLV, which additionally consists of other components such as eugenyl acetate or beta-caryophyllene. In conclusion, the antibacterial activity of CLV-loaded PCL-GEL nanofiber mats confirms the present fibrous structures as promising materials for antibacterial wound dressing.

In the present study, the cell viability of PCL-GEL nanofiber mats with and without CLV was investigated with NHDF cells. The results indicated that there were no significant differences (*p* < 0.05) in cell viability and morphology of the nanofiber mats in comparison to control (Figure 5 and Figure 6). Accordingly, the in vitro cell viability and morphology results demonstrated that PCL-GEL, PCL-GEL/CLV1.5, PCL-GEL/CLV3, and PCL-GEL/CLV6 nanofiber mats are biocompatible. In a related study, Tang et al. [46] produced peppermint and chamomile essential oil-loaded gelatin nanofibers. Their cytotoxicity results indicated that incorporation of peppermint and chamomile essential oil into gelatin nanofibers did not change the NIH-3T3 fibroblast cell viability [46]. In another study, Hajiali et al. [47] investigated the antibacterial activity and biocompatibility of alginate–lavender nanofibers. The cell viability of lavender-incorporated alginate nanofibers was 91%. It was stated that the addition of lavender oil did not affect the cell viability of human foreskin fibroblast (HFF-1) cells compared to the control group [47]. Similarly, Balasubramanian and Kodam [48] assessed mouse fibroblasts (NIH-3T3) on polyacrylonitrile (PAN)/lavender oil nanofibrous mats. Lavender-loaded nanofibrous mats showed non-cytotoxic behavior for NIH-3T3, which is in agreement with our results [48].

Wound healing is a complex and multiphase process that can be categorized into wounding, hemostasis, inflammation, proliferation, and maturation [49]. During this process, the formation of new tissue and wound healing rate are also affected by external factors [50]. In our study, the potential effect of CLV on wound healing was investigated by an in vitro wound-healing assay. This assay revealed that increasing the CLV concentration reduced the NHDF cell migration and proliferation, whereas the same samples had no adverse effect on the NHDF cell viability. On the other hand, after 24 h measurements, PCL-GEL/CLV1.5 values were not significantly different when compared with PCL-GEL. In the literature, a few studies have been conducted to assess the cell migration effect of EOs. For instance, the dose-dependent wound healing effect of *Eugenia dysenterica* DC leaves were reported by da Silva et al. [51] In a similar study, Léguillier et al. studied keratinocyte cell migration ability at various concentrations of *Calophyllum inophyllum* oil (0.01%, 0.1%, and 1% diluted in olive oil) [52]. Their results proved that the effective dose of EO enhances cell migration [52]. To the best of the authors’ knowledge, this is the first study on CLV-loaded PCL-GEL nanofiber mats using cultured NHDF cells to determine the wound closure rate. Therefore, the biological activity of CLV-loaded PCL-GEL nanofiber mats is promising and should be further investigated, quantifying in particular the time-dependent release of the EO and its effect on cell behavior.

## 5. Conclusions

We evaluated the antibacterial and biological activity of CLV-loaded PCL-GEL nanofiber mats on wound dressings. PCL-GEL and clove oil-loaded PCL-GEL nanofiber mats were successfully fabricated by electrospinning. Our results revealed that bead-free, uniform, and smooth fibers were obtained. However, the addition of CLV increased the average fiber diameter. The wettability study inferred that the addition of CLV decreased the contact angle value compared to PCL-GEL nanofiber mats. The FTIR spectrum suggested that the CLV component is loaded into the PCL-GEL nanofiber mats. Moreover, the cytotoxicity results revealed that the incorporation of CLV on the PCL-GEL nanofiber mats did not demonstrate a toxic effect on NHDF cell viability. Aside from this, the addition of CLV promoted the antibacterial properties against *S. aureus* and *E. coli.* In conclusion, CLV-loaded PCL-GEL nanofiber mats may have a potential in wound healing applications and can be considered as promising biomaterial for avoiding bacterial infections without using antibiotics.

## Figures and Tables

**Table 1:** Sample composition, sample label, average fiber diameter, and loading efficiency of nanofiber mats.

Sample Code	PCL (*w*/*v* %)	GEL (*w*/*v* %)	CLV (*v*/*v* %)	Average Fiber Diameter (nm)	Contact Angle(°)	Encapsulation Efficiency (EE) (%)
PCL-GEL	11.2	4.8	-	241 ± 96	37 ± 8	-
PCL-GEL/CLV1.5	11.2	4.8	1.5	285 ± 67	18 ± 3	53 ± 4
PCL-GEL/CLV3	11.2	4.8	3	300 ± 73	21 ± 4	68 ± 11
PCL-GEL/CLV6	11.2	4.8	6	305 ± 82	27 ± 5	73 ± 3
